# CPRSCA-ResNet: a novel ResNet-based model with Channel-Partitioned Resolution Spatial-Channel Attention for EEG-based seizure detection

**DOI:** 10.3389/fnins.2025.1693079

**Published:** 2025-10-29

**Authors:** Suhong Ye, Guibin Chen, Gang Li, Xueqian Shen

**Affiliations:** ^1^Psychiatry Department, The Second Hospital of Jinhua, Jinhua, China; ^2^College of Mathematical Medicine, Zhejiang Normal University, Jinhua, China

**Keywords:** epilepsy, seizure detection, electroencephalogram (EEG), convolutional neural network (CNN), attention mechanism

## Abstract

Epilepsy is a common chronic neurological disorder caused by abnormal discharges of brain neurons, characterized by transient disturbances in consciousness, motor function, behavior, or sensation. Recurrent seizures severely impair patients’ cognitive and physiological functions and increase the risk of accidental injury and premature death. Currently, clinical diagnosis of epilepsy mainly relies on manual interpretation of electroencephalogram (EEG) recordings, but traditional methods are time-consuming, labor-intensive, and susceptible to noise interference, highlighting the urgent need for efficient and accurate automated detection models. To address this, a novel Channel-Partitioned Resolution Spatial-Channel Attention (CPRSCA) mechanism was proposed in this study, and a CPRSCA-ResNet automatic seizure detection model was developed based on the ResNet-34 architecture. By incorporating fine-grained channel partitioning, multi-scale feature fusion, and multi-dimensional attention mechanisms, the proposed approach significantly enhances the precise representation of complex EEG features. Patient-dependent and patient-independent seizure detection experiments were conducted on the public CHB-MIT dataset and two local hospital datasets (JHCH and JHMCHH). The results show that, in patient-dependent experiments, the proposed model achieved accuracies of 99.12 ± 2.09%, 96.88 ± 4.64%, and 98.84 ± 1.75% on the three datasets, while in patient-independent experiments, accuracies reached 78.71 ± 13.06%, 87.15 ± 15.32%, and 89.23 ± 7.87%, respectively. These metrics consistently outperform state-of-the-art baselines, confirming the effectiveness and generalizability of the CPRSCA mechanism for automatic seizure detection. In summary, the proposed method provides an efficient, robust, and highly generalizable technical solution for auxiliary clinical diagnosis of epilepsy, with the potential to substantially reduce the burden of manual EEG interpretation and improve the diagnostic efficiency for patients with epilepsy.

## 1 Introduction

Epilepsy is recognized as a common chronic neurological disorder, caused by abnormal neuronal discharges that lead to transient dysfunction of the brain, manifesting as sudden disturbances in consciousness, behavior, sensation, or movement (Tieng et al., 2017). The disease primarily affects adults, who account for approximately 70% of cases, while children represent about 30% of the patient population (Prasanna et al., 2021). Recurrent epileptic seizures have been shown to induce long-term adverse effects on mental and cognitive functions, and may also result in loss of consciousness, injury, or even sudden death (Xiao et al., 2024; Roshan Zamir, 2016; Zhong et al., 2023; Zhang et al., 2024a). Moreover, the premature mortality rate among people with epilepsy is two to three times higher than that of the general population (Wong et al., 2023). In addition to physiological issues, patients frequently experience embarrassment and discrimination in social and psychological contexts, leading to substantial psychological distress (Zarei and Asl, 2021; Zhang X. et al., 2024). Given the profound impact of epilepsy on affected individuals, it is therefore essential that seizures are detected promptly and accurately through advanced detection schemes (Zhang et al., 2024c), which can improve patients’ quality of life, reduce the risks associated with seizures, and provide a basis for physicians to formulate personalized treatment plans (Shoeibi et al., 2021).

Electroencephalography (EEG) is widely regarded as one of the most important diagnostic tools for the evaluation of epilepsy (Supriya et al., 2023; Zarei et al., 2019). Compared with other diagnostic techniques such as computed tomography, magnetic resonance imaging, and functional magnetic resonance imaging, EEG offers lower cost, higher tolerance to patient movement, and no radiation risk, while providing high temporal resolution data (Song et al., 2022; Hosseini et al., 2021). During epileptic seizures, EEG signals are typically characterized by significant abnormal electrical activity, such as persistent spikes and spike-and-wave complexes (Patnaik and Manyam, 2008; Qiu S. et al., 2023; Qiu X. J. et al., 2023). Traditionally, neurologists have relied primarily on visual inspection of EEG waveforms and amplitudes to identify seizure events (Hu et al., 2024). However, due to the high sampling frequency and large data volume of EEG signals, considerable time and effort are required for manual analysis in clinical practice, resulting in low efficiency; in addition, the accuracy of detection can be compromised by environmental noise and other interference factors (Qiu X. J. et al., 2023; Hassan and Subasi, 2016; Shyu et al., 2023; Yuan et al., 2023; Tang et al., 2024; Wang B. et al., 2023; Zhu and Wang, 2023). Therefore, achieving efficient and accurate automatic seizure detection using EEG signals has become a critical issue that urgently needs to be addressed. Such advances not only help to alleviate the workload of clinicians and improve diagnostic efficiency, but also provide a solid foundation for timely and precise medical services for patients.

Epileptic seizure detection techniques are generally divided into two major categories: traditional machine learning methods and deep learning approaches. In conventional machine learning frameworks, the detection process typically consists of four key steps: signal acquisition, data preprocessing, feature extraction, and classification (Wang Z. W. et al., 2023), with feature extraction regarded as the core stage of the entire process (Qiu et al., 2023). However, traditional machine learning methods often face limitations in generalization when dealing with high-dimensional and complex EEG signals (Huang et al., 2023). These methods heavily rely on professional understanding of the pathophysiological mechanisms and clinical manifestations of epilepsy and require manual feature engineering and selection, thereby increasing dependence on domain expertise and posing significant challenges in capturing deep pathological features (Huang et al., 2023). As data dimensionality and sample size increase, excessive features may lead to increased computational costs and information redundancy, whereas too few features may prevent the model from capturing critical seizure patterns, thus reducing detection accuracy (Xiao et al., 2024). In contrast, end-to-end deep learning has demonstrated superior accuracy and generalization capability in seizure detection tasks due to its automatic feature learning capability (Tang et al., 2024; LeCun et al., 2015; He et al., 2025). Such networks can directly learn high-level, abstract representations from raw EEG signals, avoiding the subjectivity and limitations of traditional manual feature engineering (Thuwajit et al., 2022), and enabling more comprehensive characterization of complex spatiotemporal features. In recent years, convolutional neural networks (CNNs) and their variants have become mainstream solutions for automated seizure detection (Zhang et al., 2020). On this basis, residual connections have been utilized to effectively alleviate gradient vanishing in deep networks through skip connections, thereby improving feature propagation efficiency, while multi-scale structures employ parallel receptive fields to extract multilevel spatiotemporal information, allowing the model to simultaneously capture both local spikes and global rhythms associated with various seizure patterns (Shyu et al., 2023). Many seizure detection studies have achieved improved classification performance by adopting multi-scale feature extraction and residual structures (Hu et al., 2024; Shyu et al., 2023; Wang Z. W. et al., 2023). Additionally, to further eliminate redundancy and extract salient features, numerous studies have integrated attention mechanisms of various dimensions into seizure detection models. By dynamically assigning spatial or channel weights, these mechanisms enable the network to focus on spatiotemporal segments most relevant to seizures, thereby achieving new gains in sensitivity and accuracy (Hu et al., 2024; Tang et al., 2024; Alharthi et al., 2022; Zhao et al., 2021b). Furthermore, the synergy between multi-scale structures and multi-dimensional attention mechanisms provides the model with cross-scale and cross-dimensional information integration capabilities, markedly enhancing detection accuracy and robustness, and driving the ongoing advancement of automatic seizure detection technology.

In the field of epileptic seizure detection, research has mainly focused on two detection paradigms: patient-dependent (Xiao et al., 2024; Dash et al., 2020; Tang et al., 2024; Zhao et al., 2021a) and patient-independent approaches (Zhang et al., 2024a; Thuwajit et al., 2022; Liu et al., 2021; Zhao et al., 2022). On one hand, patient-dependent detection trains models on individualized EEG data, allowing the capture of unique neurophysiological patterns for each patient and thereby achieving higher detection accuracy (Thuwajit et al., 2022). This approach is often employed in long-term monitoring and personalized treatment of refractory epilepsy patients, and plays an important role in reducing missed and false detections. However, patient-dependent models require custom training data and model design for each subject, leading to high clinical deployment and large-scale application costs (Thuwajit et al., 2022). Furthermore, the performance of such models can be affected by changes in electrode placement, anatomical variability among individuals, and other factors, resulting in limited scalability and generalizability (Si et al., 2023). In contrast, patient-independent detection aims to build universal models that can be applied across individuals (Zhang et al., 2024a), with a central challenge of enhancing the model’s adaptability to inter-patient differences in EEG data distributions. While such models have greater universality and clinical applicability, their sensitivity and specificity are generally lower than those of patient-dependent models due to significant neurophysiological variability between subjects—particularly differences between age groups (e.g., children vs. adults) and epilepsy subtypes, which are reflected in the EEG patterns (Zhao et al., 2023). Overall, patient-dependent detection offers superior accuracy and individual adaptability, but high customization costs and limited scalability restrict its widespread clinical application. Patient-independent detection, though offering greater generalization potential and clinical value, still faces the challenge of handling inter-individual EEG variability to improve robustness and universality. Achieving a balance between these two approaches is expected to be a key direction for promoting the clinical translation of seizure detection technologies in the future.

Accordingly, a Channel-Partitioned Resolution Spatial-Channel Attention (CPRSCA) mechanism was proposed in this study as a core module based on the ResNet-34 backbone, resulting in the development of the CPRSCA-ResNet model for the purpose of capturing critical features in seizure detection and thereby improving detection accuracy. The CPRSCA mechanism adopts a refined channel partitioning strategy, wherein the input feature map is evenly divided into N mutually exclusive subsets. Each subset is independently modeled at a different spatial resolution using depthwise separable convolutions (DSC), enabling the full extraction of discriminative features across various resolutions. In addition, the model incorporates two distinct attention modules—Coordinate Attention (CA) and Squeeze-and-Excitation (SE)—to dynamically enhance salient information in both spatial and channel dimensions. To validate the effectiveness of the proposed method, patient-dependent and patient-independent seizure detection experiments were conducted on the public CHB-MIT dataset as well as two local hospital datasets, JHCH and JHMCHH.

## 2 Materials and methods

### 2.1 Dataset and preprocessing

#### 2.1.1 Dataset I

The CHB-MIT public dataset was obtained from Boston Children’s Hospital (Goldberger et al., 2000), comprising 23 EEG recordings from 22 patients with refractory epilepsy (5 males, aged 3–22 years; 17 females, aged 1.5–19 years). A total of 916 h of data were recorded, documenting 198 seizure events. The signals were acquired using the international 10–20 electrode placement system, sampled at 256 Hz with 16-bit resolution. Following the recommendations of Tsiouris et al., 18 channels consistently present across all cases were selected for analysis to reduce heterogeneity (Tsiouris et al., 2018): FP1-F7, F7-T7, T7-P7, P7-O1, FP1-F3, F3-C3, C3-P3, P3-O1, FZ-CZ, CZ-PZ, FP2-F4, F4-C4, C4-P4, P4-O2, FP2-F8, F8-T8, P8-O2, and T8-P8. Details of the patients are summarized in Table 1.

**Table 1:** Patient information of the CHB-MIT dataset.

Case	Age	Gender	# of Seizures	# of Seizure duration
1	11	Female	7	442
2	11	Male	3	172
3	14	Female	7	402
4	22	Male	4	378
5	7	Female	5	558
6	1.5	Female	10	138
7	14.5	Female	3	325
8	3.5	Male	5	919
9	10	Female	4	276
10	3	Male	7	447
11	12	Female	3	806
12	2	Female	27	1,475
13	3	Female	12	535
14	9	Female	8	109
15	16	Male	20	1992
16	7	Female	10	84
17	12	Female	3	293
18	18	Female	6	317
19	19	Female	3	236
20	6	Female	8	294
21	13	Female	4	199
22	9	Female	3	204
23	6	Female	7	424

#### 2.1.2 Dataset II

The JHCH dataset was newly collected at Jinhua Central Hospital, consisting of 24-h EEG recordings from six pediatric and neurology department patients with epilepsy. Of these, four were from the pediatric department and two from neurology, with a total of 87 recorded seizures. Patient ages ranged from 6 to 55 years. Patient details are summarized in Table 2.

**Table 2:** Patient information of the JHMCHH dataset.

Case	Age	Gender	# of Seizures	# of Seizure duration
1	6-y	Male	12	75
2	2-y	Male	7	160
3	2-m	Female	7	203
4	2-m	Female	1	98
5	3-m	Male	6	291
6	8-y	Male	2	20
7	4-y	Male	3	30
8	4-y	Male	2	30
9	3-y	Male	3	338
10	6-y	Female	8	144
11	9-y	Male	6	139

#### 2.1.3 Dataset III

The JHMCHH dataset was newly collected at Jinhua Maternal and Child Health Hospital, including 24-h EEG recordings from 11 pediatric epilepsy patients, documenting a total of 57 seizures. Patient ages ranged from 2 months to 9 years. Patient details are summarized in Table 3.

**Table 3:** Patient information of the JHCH dataset.

Case	Age	Gender	# of Seizures	# of Seizure duration
1	27-y	Female	17	909
2	55-y	Male	9	171
3	13-y	Male	6	122
4	6-y	Female	25	325
5	9-y	Female	4	224
6	8-y	Male	26	326

For both private datasets (JHCH and JHMCHH), EEG signals were acquired using a Nihon Kohden video-EEG system model 1200C, with a sampling rate of 500 Hz. Fifteen single-channel electrodes, identical to those in the public CHB-MIT dataset, were selected: Fp1, F7, F3, C3, P3, O1, Fz, Cz, Pz, Fp2, F4, C4, P4, O2, and F8. Bipolar montage processing was performed by computing the differential voltage between pairs of electrodes, resulting in 12 bipolar channels (FP1-F7, FP1-F3, F3-C3, C3-P3, P3-O1, FZ-CZ, CZ-PZ, FP2-F4, F4-C4, C4-P4, P4-O2, and FP2-F8), following the standards of the CHB-MIT dataset. To ensure sampling rate consistency across all datasets, the data were downsampled to 256 Hz, with a window length of 3 s. Finally, both interictal and ictal signals were selected as the two categories for patient-dependent seizure detection: interictal segments were labeled as negative samples, and ictal segments as positive samples. The studies involving humans were approved by Ethics Committee of Jinhua Central Hospital. The studies were conducted in accordance with the local legislation and institutional requirements. Written informed consent for participation in this study was provided by the participants’ legal guardians/next of kin.

### 2.2 Channel-Partitioned Resolution Spatial-Channel Attention

**Figure 1:**
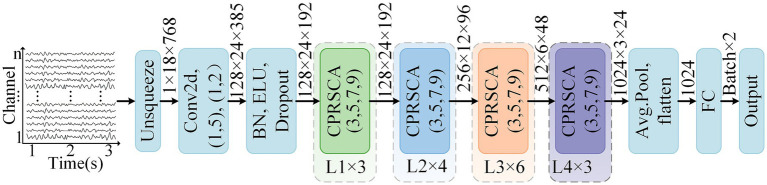
The architecture of the proposed CPRSCA-ResNet network, illustrated using 18-channel EEG signals from the CHB-MIT dataset as an example. The input EEG signals have a dimension of (18 × 768). After the Unsqueeze operation, the data are reshaped to (1 × 18 × 768), corresponding to (C × H × W). Subsequent convolutional operations are then applied, where (1, 5) and (1, 2) denote the kernel size and stride, respectively. The notation ‘L1 × 3’ indicates that the module named L1 is repeated three times, with similar conventions applied to the other layers.

**Figure 2:**
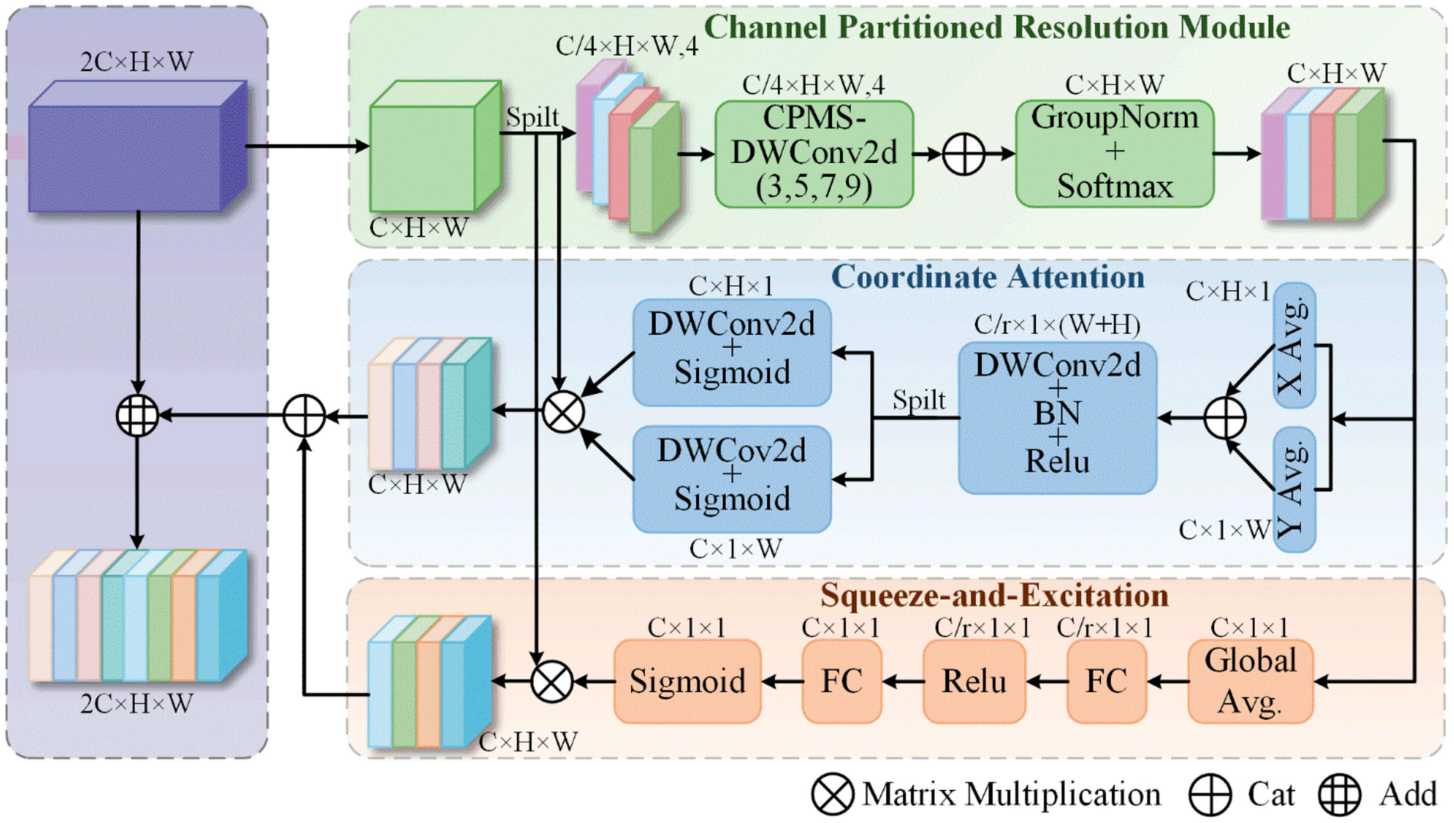
The architecture diagram of the proposed Channel-Partitioned Resolution Spatial-Channel Attention.

In this study, a novel attention mechanism termed CPRSCA was proposed based on the ResNet architecture, with ResNet-34 adopted as an illustrative example. CPRSCA was integrated as the core module of the ResNet-34 model, resulting in the CPRSCA-ResNet model, as depicted in Figure 1. The structure of the CPRSCA mechanism is illustrated in Figure 2. The code is publicly available at https://github.com/biomedicalWarehouse/CPRSCA-ResNet. In this mechanism, a fine-grained channel partitioning strategy was employed, where the input feature map was evenly divided into N mutually exclusive subsets. Each subset was independently processed at a different spatial resolution via DSCs. This hierarchical information extraction strategy enabled the model to capture features ranging from fine details to broad contextual information. After processing with DSCs, Group Normalization and the Sigmoid function were utilized for feature normalization, thereby optimizing the stability and efficiency of feature representation. In addition, CA and SE modules were incorporated to dynamically enhance salient features in both spatial and channel dimensions. This integrated attention modulation further improved the model’s ability to discriminate among complex feature networks and significantly enhanced its performance in advanced biomedical raw signal classification tasks.

#### 2.2.1 Channel-partitioned resolution module

To enable convolutional neural networks to efficiently and in parallel process convolution kernels of different sizes for capturing multi-scale information ranging from local details to broader regions and to improve computational efficiency, the proposed module first uniformly divides the input feature map $X\in {\mathbb{R}}^{C\times H\times W}$ along the channel dimension into *N* mutually exclusive subsets $X_{i}\in {\mathbb{R}}^{\frac{C}{N}\times H\times W}$ , where i∈(1,…,N) . In this study, the value of *N* was set to 4. Furthermore, to reduce the number of parameters and enhance computational efficiency, depthwise separable convolutions were adopted in place of conventional convolutions, and feature maps at different scales were extracted using convolutional kernels of sizes 3, 5, 7, and 9. After the convolutional operations, the four feature map subsets were merged to form a unified feature map $X\prime \in {\mathbb{R}}^{C\times H\times W}$ , as shown in Equation 1. Subsequently, Group Normalization was employed to normalize the merged feature map, resulting in $X\prime \prime \in {\mathbb{R}}^{C\times H\times W}$ , as presented in Equation 2. This step was designed to stabilize the feature distribution during training and enhance the generalization capability of the model. GroupNorm normalizes data by computing the mean and standard deviation within each group, independent of the batch size, and is particularly suitable for small-batch training. Finally, the Softmax function was applied to map the normalized feature values into the range [0, 1], resulting in the output feature map $Y\in {\mathbb{R}}^{C\times H\times W}$ , as shown in Equation 3. This process enhances the model’s ability to discriminate the importance of features, enabling the network to focus more on those regions that are most critical for the final task. In this way, the network is able to extract and utilize information from the feature maps more effectively, while adapting to different visual patterns and structures through the use of convolutional kernels of varying sizes, thereby improving overall recognition and processing capability.


(1) $$X\prime =\text{concat}({\left\{\text{DWConv}2d(X_{i},\text{kernel}=(i\times 2+1))\right\}}_{i=1}^{N})$$

Where DWConv2d denotes a 2D depthwise separable convolution, $X_{i}\in {\mathbb{R}}^{\frac{C}{N}\times H\times W}$ , and the value of *N* is set to 4.


(2) $$X\prime \prime =\frac{X\prime -\mu }{\sigma }\times \gamma +\beta$$

where μ denotes the mean of each group of features, σ represents the standard deviation, and γ and β are learnable scaling and shifting parameters.


(3) $$\begin{array}{c} Y=\frac{eX_{c,h,w}^{\prime \prime }}{\sum_{k=1}^{C}X_{c,h,w}^{\prime \prime }} \end{array}$$

where $X_{c,h,w}^{\prime \prime }\;$ denotes the feature value at channel *c*, height *h*, and width *w*.

#### 2.2.2 Coordinate attention

Coordinate Attention (Hou et al., 2021) enhances the spatial perception and performance of the model by encoding spatial relationships within the feature map in a precise manner through the introduction of coordinate information. This mechanism comprises two main steps: coordinate information embedding and coordinate attention generation.

##### 2.2.2.1 Coordinate information embedding

To enable the capture of long-range contextual information in the spatial domain, the input Y is subjected to average pooling along the horizontal and vertical directions for each channel, resulting in feature maps $Z^{h}$ and $Z^{w}$ , respectively. Accordingly, the output at height h for channel c can be expressed as:


(4) $$\begin{array}{c} Z_{c}^{h}(h)=\frac{1}{W}\sum_{0\leq i\leq W}Y_{c}(h,i) \end{array}$$

where $Z_{c}^{h}(h)$ denotes the output feature for channel c at height h , W is the width of the input feature map, and $Y_{c}(h,i)$ represents the feature value at position (h,i) for channel c .

Similarly, the output at width w for channel c can be written as:


(5) $$\begin{array}{c} Z_{c}^{w}(w)=\frac{1}{H}\sum_{0\leq i\leq H}Y_{c}(j,w) \end{array}$$

where $Z_{c}^{w}(w)$ indicates the output feature for channel c at width w , H is the height of the input feature map, and $Y_{c}(j,w)$ denotes the feature value at position (j,w) for channel c .

##### 2.2.2.2 Coordinate attention generation

As described above, Equations 4, 5 encode precise positional information via global receptive fields. To leverage these representations, a second transformation—coordinate attention generation—is introduced. Specifically, the aggregated feature maps obtained in Equations 4, 5 are concatenated and passed through a shared 1 × 1 convolutional transformation $F_{1}$ , yielding:


(6) $$\begin{array}{c} f=\delta (F_{1}([Z^{h},Z^{w}])) \end{array}$$

where, in Equation 6, [⋅,⋅] denotes concatenation along the spatial dimension, δ is a nonlinear activation function, and $\left\{f\in {\mathbb{R}}^{\frac{C}{r}\times (H+W)}\right\}$ is the intermediate feature map encoding both horizontal and vertical spatial information. Here, r is a reduction ratio to control the block size. The tensor f is then split along the spatial dimension into two separate tensors, $\left\{f^{h}\in {\mathbb{R}}^{\frac{C}{r}\times H}\right\}$ and $\left\{f^{w}\in {\mathbb{R}}^{\frac{C}{r}\times W}\right\}$ . Subsequently, two 1 × 1 convolutional transformations, $F_{h}$ and $F_{w}$ , are applied to $f^{h}$ and $f^{w}$ , respectively, to obtain tensors with the same number of channels as the input X , resulting in Equations 7, 8:


(7) $$\begin{array}{c} g^{h}=\sigma (F_{h}(f^{h})) \end{array}$$

(8) $$\begin{array}{c} g^{w}=\sigma (F_{w}(f^{w})) \end{array}$$

where σ denotes the sigmoid function. To reduce model complexity, an appropriate reduction ratio r (such as 16) is typically used to decrease the channel number of f . Finally, the outputs $g^{h}$ and $g^{w}$ are broadcast and used as attention weights. The final output of the coordinate attention module Y can be formulated as Equation 9:


(9) $$\begin{array}{c} Y_{c}^{\prime }(i,j)=X_{c}(i,j)\times g_{c}^{h}(i)\times g_{c}^{w}(j) \end{array}$$

Through this approach, the coordinate attention module not only captures inter-channel relationships but also enhances the model’s ability to recognize target locations by encoding spatial information.

#### 2.2.3 Squeeze-and-excitation attention

Squeeze-and-Excitation (Hu et al., 2020) is a channel attention mechanism designed to dynamically recalibrate channel-wise feature responses by learning inter-channel dependencies, thereby enhancing the model’s representational capacity while preserving the original spatial dimensions of the input feature map. This mechanism consists of two main steps: Squeeze and Excitation.

##### 2.2.3.1 Squeeze

To model the dependencies between channels, channel-wise statistics must first be obtained. The squeeze step achieves this by applying global average pooling to generate channel descriptors:


(10) $$\begin{array}{c} Z_{c}=\frac{1}{H\times W}\sum_{i=1}^{H}\sum_{j=1}^{W}Y_{c}(i,j) \end{array}$$

where, in Equation 10, $Y_{c}(i,j)$ denotes the input feature value at position (i,j) in channel c . The input feature map Y has spatial dimensions H×W and C channels. $Z_{c}$ represents the statistic for channel c .

##### 2.2.3.2 Excitation

The obtained channel descriptors are then passed through two fully connected (FC) layers and a sigmoid activation function to model the nonlinear inter-channel relationships and generate channel-wise weights:


(11) $$\begin{array}{c} S=\sigma (W_{2}*\delta (W_{1}*Z+b1)+b2) \end{array}$$

where, in Equation 11, σ denotes the sigmoid function, δ is the ReLU activation function, $\left\{W_{1}\in {\mathbb{R}}^{\frac{C}{r}\times C},b_{1}\right\}$ and $\left\{W_{2}\in {\mathbb{R}}^{\frac{C}{r}\times C},b_{2}\right\}$ represent the two fully connected layers. The reduction ratio r is employed to balance model capacity and computational complexity.

Finally, the output Y′ is obtained by channel-wise multiplication of the original input Y and the channel-wise weight vector S :


(12) $$\begin{array}{c} Y\prime =Y\cdot S \end{array}$$

where, in Equation 12, ⋅ denotes element-wise multiplication along the channel dimension. When the values in S fall within the range [0,1], the SE block is able to preserve informative features while suppressing irrelevant ones, thereby improving the overall network performance.

#### 2.2.4 Model training and evaluation

The proposed algorithm was implemented using the PyTorch deep learning framework (version 1.12). Experiments were conducted on a platform equipped with an NVIDIA GeForce RTX 3080Ti GPU (12GB VRAM) and running Windows 11. During training, a batch size of 4 was employed, and the total number of epochs was set to 50. The cross-entropy loss function was utilized, and parameter optimization was carried out using the AdamW optimizer. To enhance the training process, a dynamic learning rate adjustment strategy was adopted: the learning rate was initially set to 5 × 10^−4^ with a warm-up phase, wherein the learning rate was rapidly increased to its target value at the beginning of training, followed by a gradual decay according to a predetermined schedule. This adaptive adjustment facilitated faster convergence of model parameters toward an optimal solution in the early stages while ensuring training stability through refined learning rate decay in later stages. In terms of data partitioning, two experimental schemes were designed, namely patient-dependent and patient-independent. In the patient-dependent setting, the data of each patient were randomly divided into training and testing sets according to a 4:1 ratio, with approximately 80% of the data used for training and the remaining 20% used for testing, so as to evaluate the detection performance within the same patient. In the patient-independent setting, patient identities were strictly separated, and the Leave-One-Subject-Out strategy was adopted. Specifically, in each experiment, the entire data of one patient were designated as the testing set, while the data of all other patients were used as the training set. This procedure was iteratively rotated until every patient had served once as the testing subject, thereby providing a comprehensive evaluation of the robustness and generalization ability of the model in cross-patient scenarios. Model performance was assessed using several evaluation metrics, including accuracy, sensitivity, F1 score, and specificity, with the calculation formulas provided as Equations 13–16:


(13) $$\begin{array}{c} \text{Accuracy}=\frac{\mathrm{TP}+\mathrm{TN}}{\mathrm{TP}+\mathrm{TN}+\mathrm{FP}+\mathrm{FN}} \end{array}$$

(14) $$\begin{array}{c} \text{Sensitivity}=\frac{\mathrm{TP}}{\mathrm{TP}+\mathrm{FN}} \end{array}$$

(15) $$\begin{array}{c} F1\;\text{Score}=\frac{2\mathrm{TP}}{2\mathrm{TP}+\mathrm{FP}+\mathrm{FN}} \end{array}$$

(16) $$\begin{array}{c} \text{Specificity}=\frac{\mathrm{TN}}{\mathrm{TN}+\mathrm{FP}} \end{array}$$

## 3 Result

### 3.1 Patient-dependent seizure detection

In this study, ResNet-34 was employed as the backbone network, and a series of systematic ablation experiments were conducted to assess the performance of each component within the CPRSCA attention mechanism, as summarized in Table 4. Initially, baseline models were established by integrating multi-scale convolutional modules with SE and CA attention mechanisms, respectively. Subsequently, the proposed CPRM module was substituted for the multi-scale convolutional module, with the same attention mechanism configurations maintained for comparative analysis. The experimental results demonstrated that, under identical attention settings, the CPRM module achieved higher classification accuracy compared to the multi-scale convolutional module. These findings validate the superiority of the CPRM module in cross-scale feature fusion and highlight the synergistic enhancement effect when combined with joint spatial-channel attention mechanisms.

**Table 4:** Ablation study results for patient-dependent experiments on the three datasets.

Dataset	Methods	Accuracy (%) Mean ± std	Sensitivity (%) Mean ± std	F1 Score (%) Mean ± std	Specificity (%)Mean ± std
CHB-MIT	Baseline	98.09 ± 2.88	98.07 ± 3.00	98.05 ± 2.96	97.10 ± 4.89
	CPRM	98.22 ± 2.87	98.21 ± 2.96	98.18 ± 2.96	97.61 ± 4.78
	CPRM+CA	98.62 ± 2.77	98.65 ± 2.86	98.59 ± 2.86	97.84 ± 5.68
	CPRM+SE	93.95 ± 5.95	93.80 ± 6.10	93.77 ± 6.21	89.57 ± 11.81
	CPRM+CA + SE (CPRSCA)	**99.12 ± 2.09**	**99.12 ± 2.18**	**99.10 ± 2.14**	**98.55 ± 4.34**
JHMCHH	Baseline	94.01 ± 8.38	95.17 ± 6.31	93.84 ± 8.75	90.34 ± 12.62
	CPRM	96.32 ± 5.29	96.24 ± 5.68	96.19 ± 5.62	93.39 ± 11.52
	CPRM+CA	96.35 ± 4.32	96.66 ± 3.68	96.32 ± 4.35	94.23 ± 7.50
	CPRM+SE	96.18 ± 6.75	96.66 ± 5.72	96.17 ± 6.76	96.05 ± 8.83
	CPRM+CA + SE (CPRSCA)	**96.88 ± 4.64**	**97.23 ± 4.07**	**96.86 ± 4.67**	**96.28 ± 6.84**
JHCH	Baseline	94.63 ± 6.26	94.94 ± 5.74	94.59 ± 6.31	96.32 ± 3.82
	CPRM	96.78 ± 4.97	96.97 ± 4.58	96.76 ± 4.98	98.99 ± 1.67
	CPRM+SE	96.58 ± 4.29	96.51 ± 4.44	96.53 ± 4.36	93.90 ± 7.87
	CPRM+CA	97.01 ± 2.90	96.97 ± 3.07	96.97 ± 2.96	96.22 ± 5.98
	CPRM+CA + SE (CPRSCA)	**98.84 ± 1.75**	**98.93 ± 1.61**	**98.83 ± 1.76**	**99.24 ± 1.69**

Bold value indicates the optimal result.

**Figure 3:**
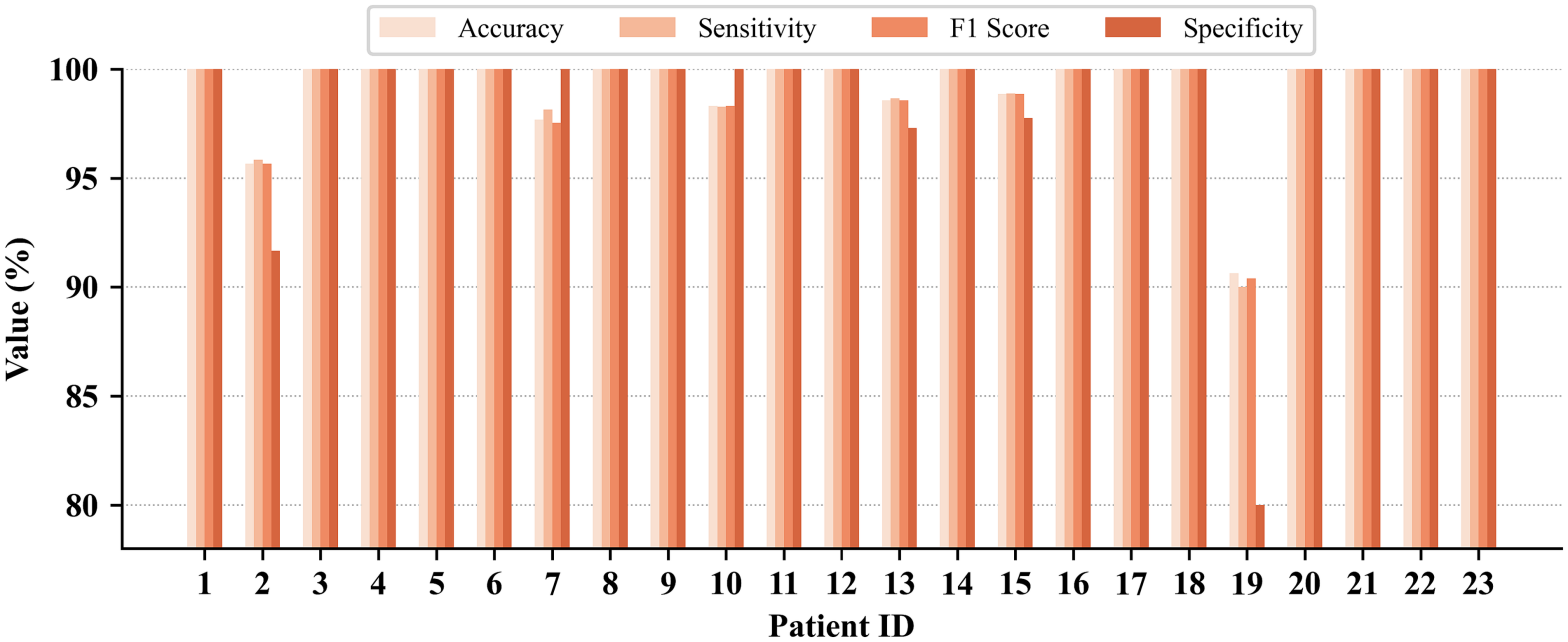
Patient-dependent seizure detection results on the CHB-MIT dataset.

**Figure 4:**
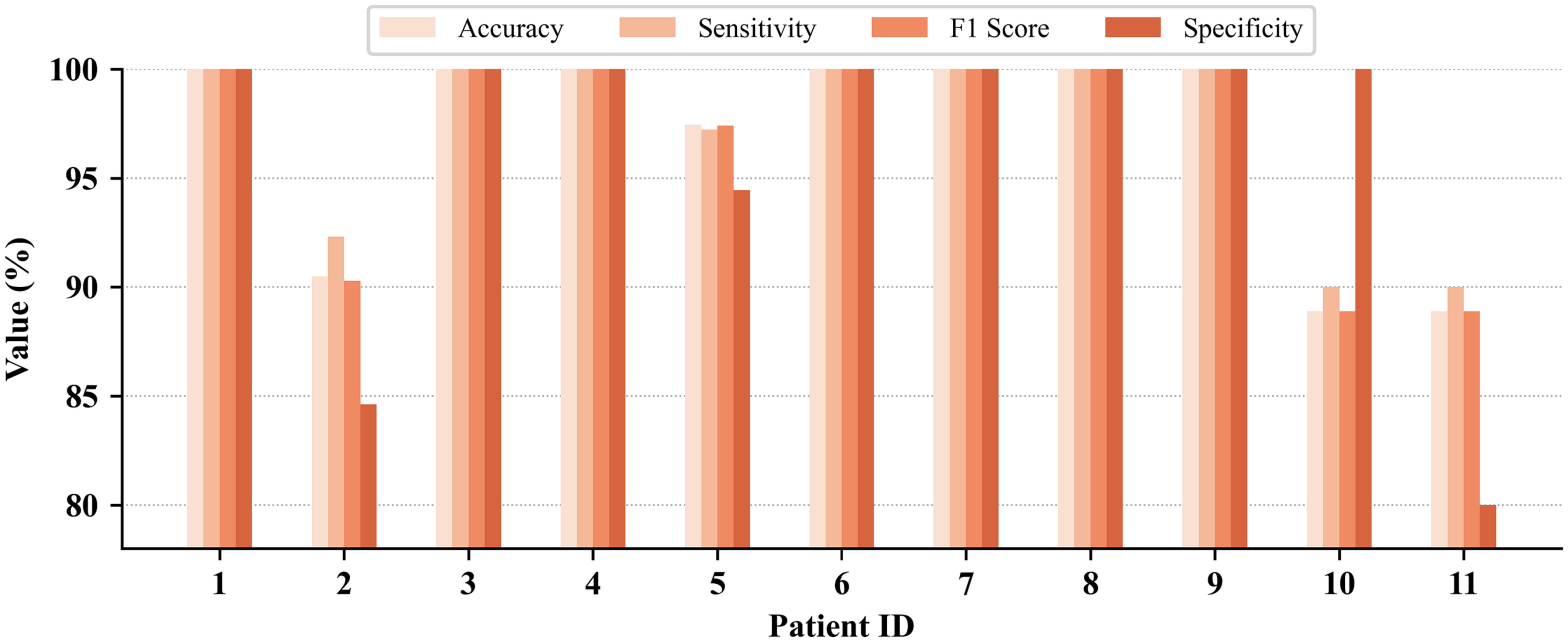
Patient-dependent seizure detection results on the JHMCHH dataset.

**Figure 5:**
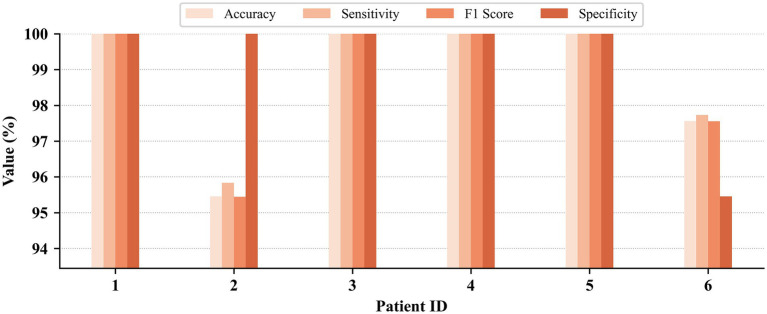
Patient-dependent seizure detection results on the JHCH dataset.

Patient-dependent seizure detection results on the CHB-MIT, JHMCHH, and JHCH datasets are presented in Figures 3–5, respectively. On the CHB-MIT dataset, the performance for Accuracy, Sensitivity, F1 Score, and Specificity was observed to be 99.12 ± 2.09%, 99.12 ± 2.18%, 99.10 ± 2.14%, and 98.55 ± 4.34%, respectively. On the JHMCHH dataset, the corresponding metrics were 96.88 ± 4.64%, 97.23 ± 4.07%, 96.86 ± 4.67%, and 96.28 ± 6.84%. On the JHCH dataset, the performance for these four metrics reached 98.84 ± 1.75%, 98.93 ± 1.61%, 98.83 ± 1.76%, and 99.24 ± 1.69%, respectively. It should be noted that lower scores in some patients may be attributed to relatively short seizure durations in EEG recordings and the presence of considerable noise in the data.

### 3.2 Patient-independent seizure detection

Ablation experiments for patient-independent seizure detection were first conducted to assess the performance of the CPRSCA model, as presented in Table 5. The multi-scale module was initially adopted as the baseline model, followed by an evaluation of the classification performance when combined with CA, SE, and their combination. Further assessments were carried out by integrating the CPRM module with CA and SE individually, as well as by incorporating multi-scale techniques together with CA and SE, and the results were compared with those of the multi-scale module. As observed in Table 5, the integration of the CPRM module with the two distinct attention mechanisms resulted in a further improvement in accuracy, indicating that the complementary effects among these modules collectively enhance patient-independent seizure detection performance.

**Table 5:** Ablation study results for patient-independent experiments on the three datasets.

Dataset	Methods	Accuracy (%) Mean ± std	Sensitivity (%) Mean ± std	F1 Score (%) Mean ± std	Specificity (%) Mean ± std
CHB-MIT	Baseline	76.26 ± 17.24	76.26 ± 17.21	74.36 ± 19.91	77.18 ± 23.24
	CPRM	76.48 ± 16.89	76.46 ± 16.89	75.07 ± 18.81	74.32 ± 23.11
	CPRM+CA	77.38 ± 15.05	77.36 ± 15.07	75.54 ± 17.62	79.00 ± 21.26
	CPRM+SE	77.20 ± 13.00	77.17 ± 13.01	75.06 ± 16.79	76.64 ± 23.20
	CPRM+CA + SE (CPRSCA)	**78.71 ± 13.06**	**78.70 ± 13.04**	**77.75 ± 14.53**	**79.16 ± 16.80**
JHMCHH	Baseline	84.83 ± 14.57	84.63 ± 14.71	82.68 ± 18.82	74.49 ± 29.28
	CPRM	86.06 ± 15.41	85.92 ± 15.59	83.91 ± 19.69	77.07 ± 30.57
	CPRM+CA	86.82 ± 13.73	86.64 ± 13.86	85.27 ± 16.74	78.89 ± 26.48
	CPRM+SE	86.84 ± 13.52	86.73 ± 13.72	85.19 ± 17.02	78.96 ± 29.17
	CPRM+CA + SE (CPRSCA)	**87.15 ± 15.32**	**87.03 ± 15.48**	**85.32 ± 18.65**	**79.10 ± 30.27**
JHCH	Baseline	85.81 ± 7.30	85.74 ± 7.38	85.40 ± 7.58	76.29 ± 16.80
	CPRM	88.93 ± 6.89	88.87 ± 6.95	88.60 ± 7.23	82.83 ± 15.44
	CPRM+CA	89.09 ± 8.28	89.02 ± 8.35	88.74 ± 8.63	79.78 ± 17.19
	CPRM+SE	89.06 ± 6.64	89.04 ± 6.65	89.04 ± 6.65	84.87 ± 15.03
	CPRM+CA + SE (CPRSCA)	**89.23 ± 7.87**	**89.18 ± 7.92**	**88.99 ± 8.11**	**81.39 ± 15.53**

Bold value indicates the optimal result.

**Figure 6:**
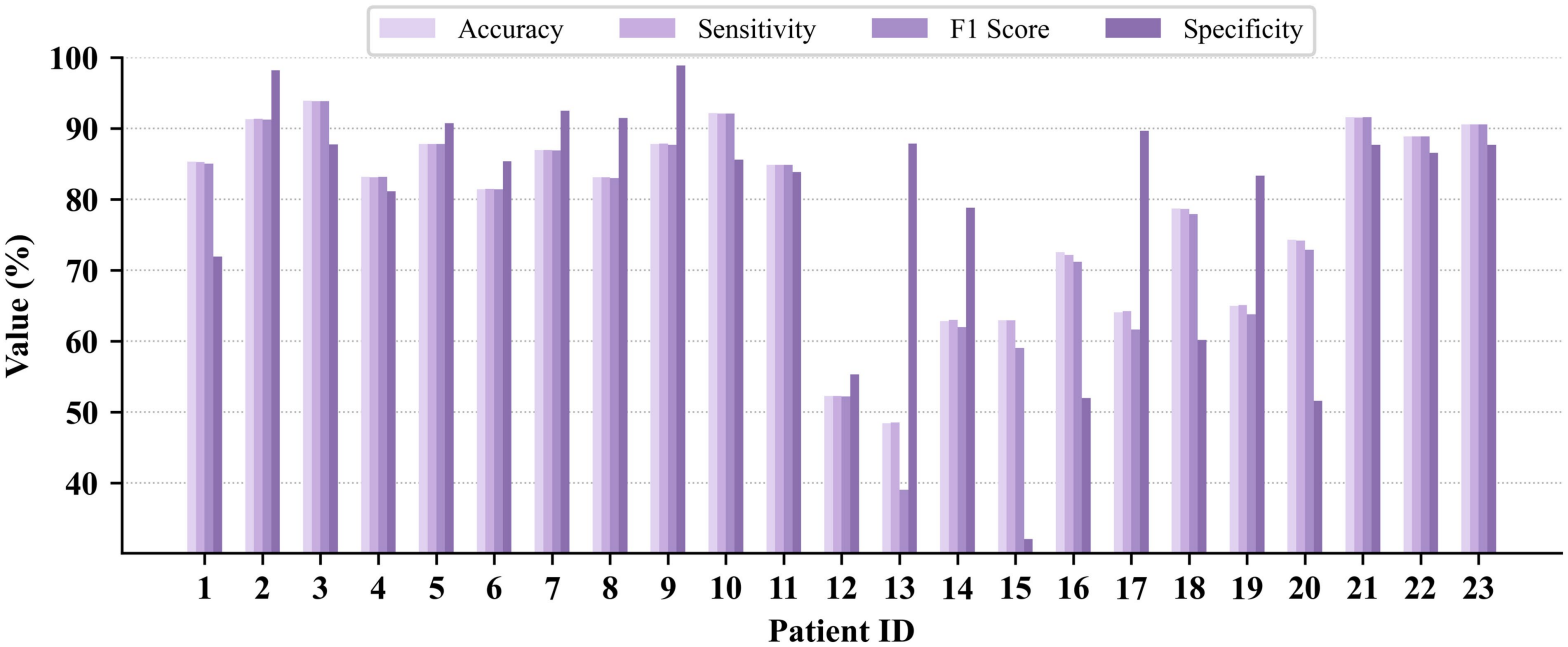
Patient-independent seizure detection results on the CHB-MIT dataset.

**Figure 7:**
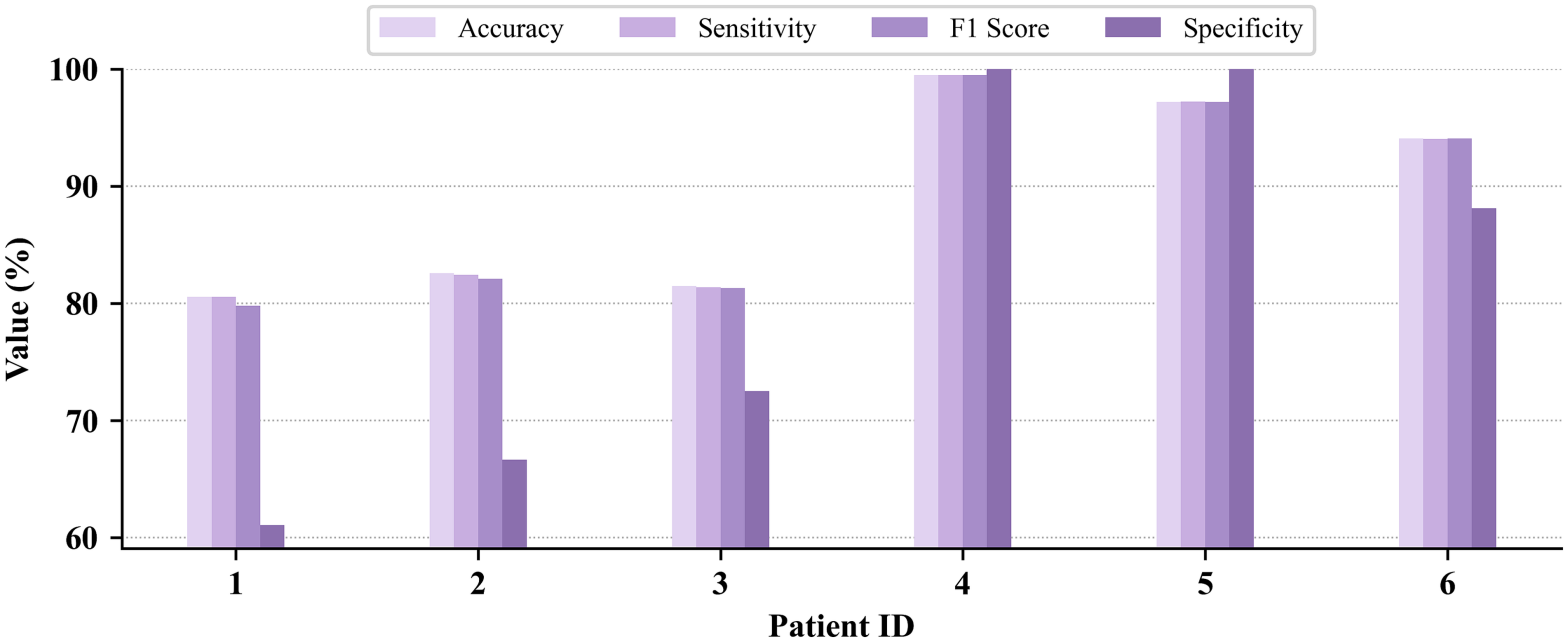
Patient-independent seizure detection results on the JHMCHH dataset.

**Figure 8:**
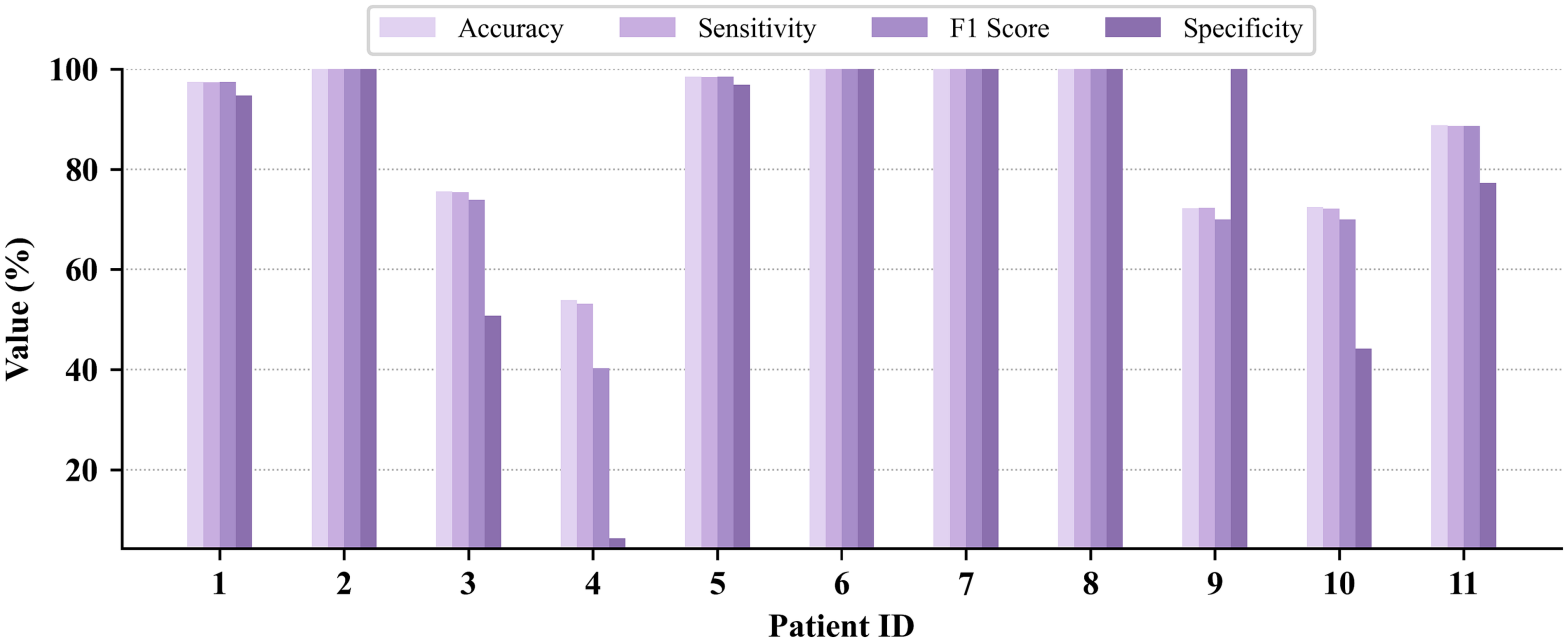
Patient-independent seizure detection results on the JHCH dataset.

Patient-independent seizure detection results on the CHB-MIT, JHMCHH, and JHCH datasets are illustrated in Figures 6–8, respectively. On the CHB-MIT dataset, Accuracy, Sensitivity, F1 Score, and Specificity were observed to be 78.71 ± 13.06%, 78.70 ± 13.04%, 77.75 ± 14.53%, and 79.16 ± 16.80%, respectively. On the JHMCHH dataset, these metrics were recorded as 87.15 ± 15.32%, 87.03 ± 15.48%, 85.32 ± 18.65%, and 79.10 ± 30.27%. On the JHCH dataset, the results for the four metrics were 89.23 ± 7.87%, 89.18 ± 7.92%, 88.99 ± 8.11%, and 81.39 ± 15.53%, respectively.

## 4 Discussion

A novel CPRSCA attention mechanism was proposed in this study and integrated as the core module of the ResNet-34 model, demonstrating successful application to EEG-based seizure detection tasks. The principal innovation of the CPRSCA mechanism lies in its CPRM module, which adopts a channel-partitioned multi-scale feature extraction strategy to effectively integrate salient EEG information across different spatial resolutions. Furthermore, by combining channel attention (SE) and spatial attention (CA) mechanisms, the model is able to dynamically enhance its focus on features relevant to epileptic seizures. Experimental validation was conducted on both the public CHB-MIT dataset and two local hospital datasets (JHCH and JHMCHH) under patient-dependent and patient-independent protocols, where the proposed approach consistently exhibited superior detection performance.

Compared with conventional single-scale or single-dimension attention mechanisms, the proposed CPRSCA mechanism significantly improved the model’s sensitivity and robustness in recognizing seizure patterns. Specifically, the ablation studies presented in Tables 4, 5 revealed that the CPRM module played a critical role in enhancing feature representation, effectively reducing redundant information and noise through multi-scale channel partitioning, and substantially improving generalization across different patients and acquisition devices. Moreover, when either channel or spatial attention was used in isolation, model performance declined to varying degrees, further demonstrating the clear advantage of multidimensional attention fusion strategies in improving model robustness. Previous studies have reported that the fusion of channel and spatial attention mechanisms in seizure detection leads to certain performance improvements (Wang Z. W. et al., 2023; Zhong et al., 2025; He et al., 2022; Zhang et al., 2023; Li et al., 2020). In the present study, a multi-scale channel partitioning strategy was further introduced, allowing for a more thorough extraction of discriminative EEG features at different scales (Thuwajit et al., 2022), thereby enabling further performance gains atop multidimensional attention mechanisms. In addition, DSCs were employed in place of conventional convolutions, resulting in a reduction in model parameters while maintaining performance (Si et al., 2023). The collective design of these architectural elements highlights the advancement of the CPRSCA mechanism and offers new theoretical perspectives and technical pathways for EEG analysis and seizure detection.

Moreover, the practical potential of the CPRSCA mechanism was demonstrated under both patient-dependent and patient-independent experimental settings. In the patient-dependent seizure detection experiments, the proposed method exhibited high levels of accuracy, sensitivity, and specificity across all three datasets, particularly adapting well to individual neurophysiological differences in the locally collected clinical datasets. This indicates the strong practical value of the CPRSCA mechanism in clinical practice, with promise for supporting personalized and precise monitoring and treatment of refractory epilepsy patients. Additionally, an in-depth comparison was performed on the public CHB-MIT dataset with recent related studies, as shown in Table 6. Compared with these advanced seizure detection methods, the proposed approach achieved clear advantages in all performance metrics, especially in terms of accuracy and sensitivity, highlighting the unique capability of the CPRSCA mechanism to capture individualized EEG features. Furthermore, in the more challenging patient-independent experiments (see Table 7 for comparison with other advanced methods), superior performance was achieved across all evaluation metrics compared to current mainstream detection models, further confirming the effectiveness of the model in addressing inter-individual variability and enhancing generalizability. The patient-independent approach, by training a unified model for all subjects, is designed to capture generalized seizure characteristics across individuals, which offers greater applicability for real clinical scenarios and can further improve the work efficiency of clinicians (Zhang et al., 2024a). However, due to significant EEG variability among subjects, the overall performance of patient-independent detection typically remains lower than that of patient-dependent approaches (Hu et al., 2024; Zhao et al., 2023). This discrepancy is primarily attributed to the brevity of individual seizure patterns, the high heterogeneity of neurophysiological features, and the prevalence of noise in the data (Shyu et al., 2023). Therefore, future research should focus on further reducing the impact of inter-individual differences on detection performance and enhancing the robustness and practicality of the model in complex clinical environments. In summary, the proposed CPRSCA attention model not only exhibited outstanding performance in seizure detection tasks across diverse data sources, but also demonstrated broad prospects for application in both personalized and generalized seizure monitoring and auxiliary diagnosis in real clinical environments. This capability has the potential to reduce clinicians’ workload and improve the efficiency of auxiliary diagnosis.

**Table 6:** Comparison of patient-dependent seizure detection results between the proposed method and other research methods on the CHB-MIT dataset.

Authors	Type	Method	Accuracy (%)	Sensitivity (%)	F1 Score (%)	Specificity (%)
Zhang et al. (2024b)	Machine Learning	DLFE	98.63	98.06	99.00	99.19
Wang et al. (2024)		Decision Tree	96.00	92.7	–	97.6
Zabihi et al. (2016)		LDA	94.69	89.10	–	94.80
Zhang et al. (2022)	Deep Learning	Bi-GRU	98.49	93.89	–	98.49
Tang et al. (2024)		PS + Bi-LSTM + Attentions	99.09	99.28	98.89	98.95
Sun et al. (2024)		MDFLN+BLSTM	97.08	97.53	–	96.63
Thuwajit et al., 2022	End-to-End Deep Learning	EEGWaveNet	98.39	68.94	98.01	99.25
Shyu et al. (2023)		Inception and Residual model	98.34	73.08	69.34	98.79
Zhao et al. (2023)		HAN	98.30	97.34	96.46	96.07
Liu et al. (2025)		group CosCNN	97.54	97.70	–	97.54
Zhao et al. (2021b)		GAT	98.89	97.10	–	99.63
Hu et al. (2024)		Inresformer	98.03	95.65	–	98.01
Wang et al. (2025)		CNN-ViT	98.00	99.34	–	98.01
Our method		CPRSCA-ResNet	99.12	99.12	99.10	98.55

**Table 7:** Comparison of patient-independent seizure detection results between the proposed method and other research methods on the CHB-MIT dataset.

Authors	Method	Accuracy (%)	Sensitivity (%)	F1 Score (%)	Specificity (%)
Zhao et al. (2023)	HAN	73.15	72.75	73.77	75.70
Xiao et al. (2024)	SSL	74.67	77.10	76.61	78.36
Zhao et al. (2022)	MVCO&IBA	76.36	76.42	76.11	76.32
Zhang et al. (2022)	Bi-GRU	79.79	58.25	–	79.97
This Work	CPRSCA-ResNet	78.71	81.49	79.56	83.88

Despite the promising performance achieved by the proposed CPRSCA mechanism in seizure detection tasks, several limitations remain. First, due to substantial heterogeneity introduced by patient-dependent characteristics, age groups, and seizure types, the detection accuracy for certain patients requires further improvement, which places higher demands on the model’s generalization and robustness. Second, although the integration of the CPRSCA mechanism with the ResNet-34 backbone—along with the adoption of DSCs—effectively reduces the number of parameters, the overall model architecture remains relatively complex, and computational efficiency in resource-constrained clinical settings still needs enhancement. Finally, experimental validation in this study was primarily conducted on offline data, and prospective bedside monitoring studies have not yet been performed in real clinical environments. In future work, it is suggested that the dataset be expanded to cover a wider range of age groups and epilepsy types, the model architecture be further optimized and simplified, and the capability for automatic extraction of critical features and real-time detection be improved. Moreover, prospective validation should be conducted in actual clinical settings to comprehensively assess the clinical applicability and value of the proposed model.

## 5 Conclusion

In this study, a novel CPRSCA multidimensional attention mechanism was designed, and a CPRSCA-ResNet model for seizure detection was developed based on the ResNet-34 architecture. By employing fine-grained channel partitioning and multi-scale feature fusion strategies, the model enabled accurate capture and enhancement of key seizure-related features in EEG signals, resulting in significant improvements in detection performance and generalization capability. Patient-dependent and patient-independent seizure detection experiments were conducted on the public CHB-MIT dataset and two local hospital datasets (JHCH and JHMCHH), all of which yielded excellent detection results and further validated the effectiveness of the CPRSCA mechanism. Owing to its efficient and robust detection performance, the CPRSCA-ResNet model is expected to substantially reduce the burden of manual EEG interpretation by clinicians, effectively lower the risk of missed detections, and provide real-time, accurate, and individualized diagnostic support for epilepsy patients in clinical practice. Overall, this approach offers an efficient and reliable technical solution for clinical seizure monitoring and auxiliary diagnosis, demonstrating high practical value and broad prospects for clinical application, with the potential to significantly improve the efficiency and quality of epilepsy management.

## Data Availability

The datasets presented in this study can be found in online repositories. The names of the repository/repositories and accession number(s) can be found in the article/supplementary material.
